# Heat transfer analysis of Oldroyd–B nanofluid flow over a horizontal plate

**DOI:** 10.1371/journal.pone.0317297

**Published:** 2025-04-17

**Authors:** Ruishi Liang, Hanifa Hanif, Jie Song, Rahimah Mahat

**Affiliations:** 1 School of Mathematics and Statistics, Shaoguan University, Shaoguan, Guangdong, China; 2 Department of Mathematics, Sardar Bahadur Khan Women’s University, Quetta, Pakistan; 3 Universiti Kuala Lumpur Malaysian Institute of Industrial Technology, Masai, Malaysia; University of Science and Technology of China, CHINA

## Abstract

Non–Newtonian fluids have grown in popularity across a wide range of engineering disciplines. Generalized Oldroyd–B fluids are a type of non–Newtonian fluid that may mimic the behavior of many dilute polymeric liquids. On the other hand, heat transmission is important because it has industrial applications. The use of nanofluids, which have a higher heat transfer capacity, can enhance the overall efficiency of the thermal system. Therefore, this research considers the generalized Oldroyd–B nanofluid over a horizontal plate. The nonlinear fractional model is solved using the finite difference method. The integer order derivatives are integrated using the Crank–Nicolson method whereas the time fractional derivatives are evaluated using the Caputo derivative. The simulations are carried out in MATLAB software. The results revealed that the retardation time parameter slow downs the fluid. The heat transfer rates increased with increasing values of the nanoparticle volume fraction. The heat transfer of regular fluid increased by 9.4% on adding nanoparticles.

## Introduction

Over the past century, nanotechnology has gained widespread recognition as a field of research [1]. Numerous novel materials and gadgets with applications in nanomedicine, nanoelectronics, biomaterials, energy generation, and consumer goods may be created using nanotechnology. The dispersion of nanoparticles in a base fluid has been suggested to modify the fluid’s properties to maximize its thermal performance; Choi and Eastman first presented this concept [2,3]. The resultant fluid is known as a nanofluid in the literature. Nanofluids have several uses in drug delivery, heating/cooling, electronics, solar energy, food processing, etc. The use of nanofluid in automobile cooling systems was proposed by Choi [4]. A nanofluid’s efficiency and application depend on the nanoparticles floating in the base fluid. Nanoparticles are particulate substances with a minimum size of 100 nm [5]. Depending on their physical and chemical properties, nanoparticles can be categorized into several classes such as metal, metal oxide, graphite, carbon nanotubes, etc [6]. Furthermore, one of the frequent problems in a variety of sectors, including manufacturing, heavy machinery and engines, electronics, and other industries, is heat transmission. Conventional heat transfer fluid has some drawbacks; these restrictions can be removed by nanofluid through enhanced thermophysical characteristics [7]. Heat transfer and entropy generation in nanofluid within a pipe were examined by Qin [8]. The obtained results showed that the Nusselt number augments about 18.3% as the width ratio changes from 0.25 to 0.75. Furthermore, the novel nanofluid has superior thermal properties compared to water. Turkyilmazoglu [9] found that using a single–phase model resulted in reliable thermal prospects and outputs for nanofluid flow. Hanif et al. [10,11] discussed how can nanoparticles optimize the heat transfer and entropy generation of fluid. Du et al. [12] conducted an experimental study and used water–based CuO nanofluid to examine the thermal performance of a geothermal heat exchanger. They discovered that using nanofluid boosted the geothermal heat exchanger system’s pumping power consumption and heat transfer rate by 16.75% and 39.84%, respectively.

The researchers are now investigating the flow of non–Newtonian fluids from various perspectives. Non–Newtonian fluids present distinct challenges and opportunities in many applications, while Newtonian fluids are well understood [13]. The most common applications of non–Newtonian fluid include molten polymers, milk, ketchup, shampoo, paint, grease, drilling fluid, unusual lubricants, and polymer solutions [14]. Non–Newtonian fluids have unique properties that cannot be described by a single constitutive relation [15]. Non–Newtonian fluids’ versatility drives innovation across various industries [16,17]. The Maxwell fluid is the simplest subclass of rate–type fluids [18]. It predicts just stress relaxation effects, not stress retardation effects. To address this shortcoming, an Oldroyd–B fluid model was developed to study stress relaxation and retardation effects. The Oldroyd–B fluid model also provides the greatest description of the behavior of viscoelastic materials, especially the reaction of various polymeric liquids. These fluids also experience the memory and elastic effects that most biological liquids and polymers do, which makes them highly valuable in the chemical and process industries [19]. Heat transfer and flow of non–linear Oldroyd–B over a stretching cylinder were studied by Yasir and Khan [20]. They found that the heat transfer rate increased when the prescribed surface temperature was replaced with a constant wall temperature. Jyoti et al. [21] discussed the 3D flow of Oldroyd–B fluid subject to the activation energy. They claimed that the average kinetic energy of fluid particles increases in direct proportion to the radiation parameter values. Increased kinetic energy leads to more frequent and intense collisions between fluid particles, which raises the fluid temperature. Dadheech et al. [22] examined the effects of melting, slip, angled magnetic field, and chemical reactions on Oldroyd–B fluid flow across a permeable surface. Gope et al. [23] examined heat transfer and entropy generation in Oldroyd–B with a hybrid nanostructure on a radially stretched surface. According to their results, augmented relaxation and retardation time parameters control the rate of heat transmission. Furthermore, entropy generation is minimized against relaxation and retardation parameters. Sun et al. [24] investigated the instability features of the Oldroyd–B fluid with non–Fourier heat flux using the Chebyshev collocation method. The results revealed that the non–Fourier effect and relaxation time help to destabilize the system for oscillatory convection. They also claimed that the retardation time might reduce the instability of the convective system. Rathore and Sandeep [25] investigated the viscoelastic nature of blood using Maxwell and Oldroyd–B hybrid nanomodels. Their results revealed that the flow of GO–Al_2_O_3_ suspended blood using the Oldroyd–B model results in a faster heat transfer rate than the Maxwell model. Additionally, medication resistance is negligible in Oldroyd–B flow.

This work aims to give a numerical analysis of heat transfer of Oldroyd–B nanofluid flow over a horizontal plate. The considered nanofluid is the mixture of Al_2_O_3_ and mineral oil. An unconditionally stable numerical strategy based on the L1 algorithm and Crank Nicolson technique is used to achieve the numerical solutions. Examining how regulating factors affect fluid characteristics, the findings are visually shown and thoroughly described. The impacts of controlling factors on fluid characteristics are investigated, and the findings are graphically shown and explained in depth.

## Mathematical formulation

The following constitutive relation predicts the rheology of an Oldroyd–B fluid.


$$\begin{array}{ccc} T=-PI+S, & & \end{array}$$ (1)

where *P*: hydro–static pressure, *I*: identity matrix, *S*: extra stress tensor given as


$$\begin{array}{ccc} (1+\lambda_{1}D_{t})S=\mu (1+\lambda_{2}D_{t})A_{1}. & & \end{array}$$ (2)

Here *μ* denotes viscosity, *λ*_1_ is relaxation time, $\lambda_{2}(<\lambda_{1})$ is retardation time, $A_{1}=\nabla V\;+\;{\left(\nabla V\right)}^{†}$ denotes Rivlin–Erickson tensor, †indicates transpose operation and the *D* represents Oldroyd derivative defined as


$$\begin{array}{ccc} D_{t}S=\frac{\partial S}{\partial t}+V.\nabla S-(\nabla V)S-S(\nabla V{)}^{†}. & & \end{array}$$ (3)

**Fig 1:**
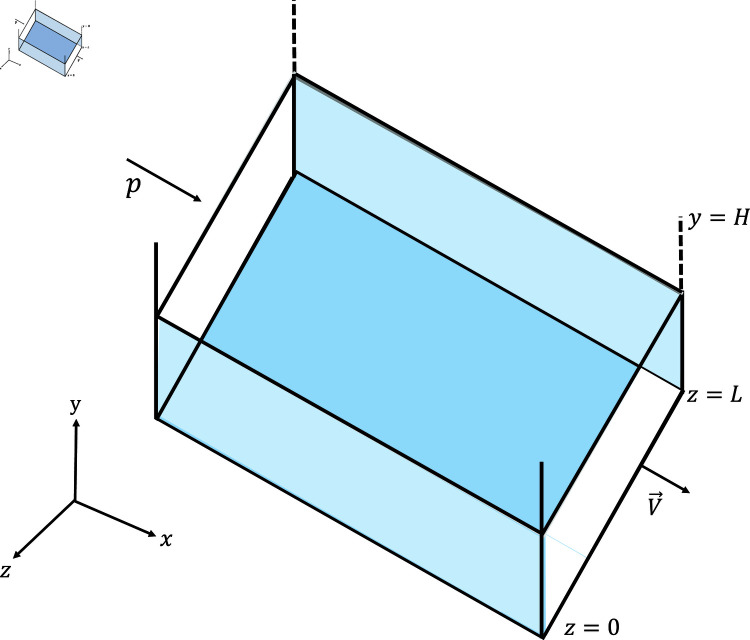
Graphical representation.

Let the Oldroyd–B nanofluid flow over a plate parallel to *xz*–plane, shown in Fig 1. The heat transfer of an Oldroyd–B nanofluid flow is characterized by continuity, momentum, and energy equations [26,27]:


$$\begin{array}{ccc} \nabla \cdot V=0, & & \end{array}$$ (4)

$$\begin{array}{ccc} \rho_{nf}(\frac{\partial V}{\partial t}+(V\cdot \nabla )V)=\nabla \cdot T, & & \end{array}$$ (5)

$$\begin{array}{ccc} (\rho C_{p}{)}_{nf}(\frac{\partial T}{\partial t}+(V\cdot \nabla )T)=k_{nf}\Delta T+T:\nabla V, & & \end{array}$$ (6)

here $\rho ,C_{p},k$, and *T* represent the density, heat capacity, thermal conductivity, body force, and temperature, respectively.

Let (*e_x_,e_y_,e_z_*) be the canonical basis for ℝ, then the velocity field is defined as


$$\begin{array}{ccc} V=ue_{x}+ve_{y}+we_{z}. & & \end{array}$$ (7)

Because the mainstream flow occurs along the *x*–axis, and *v* = 0 = *w*, we arrived at


$$\begin{array}{ccc} V=u(y,z,t)e_{x}, & & \end{array}$$ (8)

and the extra stress tensor is


$$\begin{array}{ccc} S=S(y,z,t). & & \end{array}$$ (9)

For the Oldroyd–B nanofluid with the velocity field (8), the relation (2) with fractional time derivative yield us


$$\begin{array}{ccc} & (1+\lambda_{1}^{\alpha }\frac{\partial^{\alpha }}{\partial t^{\alpha }})\tau_{xy}=\mu_{nf}\;(1+\lambda_{2}^{\beta }\frac{\partial^{\beta }}{\partial t^{\beta }})\frac{\partial u}{\partial y}, & \end{array}$$ (10)

$$\begin{array}{ccc} & (1+\lambda_{1}^{\alpha }\frac{\partial^{\alpha }}{\partial t^{\alpha }})\tau_{xz}=\mu_{nf}\;(1+\lambda_{2}^{\beta }\frac{\partial^{\beta }}{\partial t^{\beta }})\frac{\partial u}{\partial z}. & \end{array}$$ (11)

Using Eqs (1) to (3) and (8) to (11) in governing Eqs (4) to (6) yields us to


$$\begin{array}{ccc} \rho_{nf}\;(1+\lambda_{1}^{\alpha }\frac{\partial^{\alpha }}{\partial t^{\alpha }})\frac{\partial u}{\partial t}=-(1+\lambda_{1}^{\alpha }\frac{\partial^{\alpha }}{\partial t^{\alpha }})\frac{\partial P}{\partial x}+\mu_{nf}\;(1+\lambda_{2}^{\beta }\frac{\partial^{\beta }}{\partial t^{\beta }})(\frac{\partial^{2}u}{\partial y^{2}}+\frac{\partial^{2}u}{\partial z^{2}}). & & \end{array}$$ (12)

$$\begin{array}{c} (\rho C_{p}{)}_{nf}\frac{\partial T}{\partial t}=k_{nf}\;(\frac{\partial^{2}T}{\partial y^{2}}+\frac{\partial^{2}T}{\partial z^{2}})-\frac{\partial q_{r}}{\partial y}+\tau_{xy}\frac{\partial u}{\partial y}+\tau_{xz}\frac{\partial u}{\partial z}. \end{array}$$ (13)

The considered initial and boundary conditions are


$$\begin{array}{ccc} \begin{array}{ccc} & u(y,z,0)=0,\;T(y,z,0)=T_{\infty },\;\forall (y,z), & \\ & u(0,z,t)=0,\;\frac{\partial T(0,z,t)}{\partial y}=-hT,\;t>0,\;z\in [0,L],\\ & u(y,0,t)=0,\;T(y,0,t)=T_{\infty },\;t>0,\;y\in [0,\infty ),\\ & u(y,L,t)=0,\;T(y,L,t)=T_{\infty },\;t>0,\;y\in [0,\infty ),\\ & u(y,z,t)\to 0,\;T(y,z,t)\to T_{\infty },\;t>0,\;y\to \infty ,\;z\in [0,L]. \end{array} & & \end{array}$$ (14)

Further, we assume that a pressure gradient is applied at time *t* = 0 and then maintained for a time interval [0 *t*], defined as


$$\begin{array}{ccc} \frac{1}{\rho_{f}}\frac{\partial P}{\partial x}=p_{0}H(t), & & \end{array}$$ (15)

where *p*_0_ is the constant pressure gradient and *H* ( *t* )  is the Heaviside step function defined by:


$$\begin{array}{ccc} H(t)=\{\begin{array}{c} 1,\;t>0,\;\\ 0,\;t<0.\; \end{array} & & \end{array}$$ (16)

In Eq (13), *q_r_* is the radiative heat flux and defined as [27]


$$\begin{array}{ccc} q_{r}=-\frac{16\sigma_{b}T_{\infty }^{3}}{3k_{b}}\frac{\partial T}{\partial y}. & & \end{array}$$ (17)

Now, let us introduce the following set of non–dimensional variables is introduced:


$$\begin{array}{ccc} \begin{array}{c} ȳ=\frac{y}{L},\bar{z}=\frac{z}{L},\bar{t}=\frac{\nu_{f}t}{L^{2}},ū=\frac{uL}{\nu_{f}},\bar{T}=\frac{T-T_{\infty }}{T_{\infty }},\bar{p}=\frac{p_{0}L^{3}}{\nu_{f}^{2}}. \end{array} & & \end{array}$$ (18)

By using (18), after dropping bars for brevity and the adoption of the same notation for non-dimension quantities, Eqs (12) and (13) become:


$$\begin{array}{ccc} \chi_{1}(1+\lambda_{1}^{\alpha }\frac{\partial^{\alpha }}{\partial t^{\alpha }})\frac{\partial u}{\partial t}=p_{0}(H(t)+\frac{\lambda_{1}^{\alpha }t^{-\alpha }}{\Gamma (1-\alpha )})+\chi_{2}(1+\lambda_{2}^{\beta }\frac{\partial^{\beta }}{\partial t^{\beta }})(\frac{\partial^{2}u}{\partial y^{2}}+\frac{\partial^{2}u}{\partial z^{2}}). & & \end{array}$$ (19)

$$\begin{array}{ccc} \chi_{3}Pr\frac{\partial T}{\partial t}=(\chi_{4}+Rd)\frac{\partial^{2}T}{\partial y^{2}}+\chi_{4}\frac{\partial^{2}T}{\partial z^{2}}+E(\tau_{xy}\frac{\partial u}{\partial y}+\tau_{xz}\frac{\partial u}{\partial z}). & & \end{array}$$ (20)

Here


$$\begin{array}{ccc} \begin{array}{c} \chi_{1}=\frac{\rho_{nf}}{\rho_{f}},\;\chi_{2}=\frac{\mu nf}{\mu_{f}},\;\chi_{3}=\frac{(\rho C_{p}{)}_{nf}}{(\rho C_{p}{)}_{f}},\;\chi_{4}=\frac{k_{nf}}{k_{f}},\;Pr=\frac{(C_{p}\mu {)}_{f}}{k_{f}},\;Rd=\frac{16\sigma_{b}T_{\infty }^{3}}{3k_{b}},\;E=\frac{\mu_{f}\nu_{f}^{2}}{k_{f}T_{\infty }L^{3}}. \end{array} & & \end{array}$$ (21)

The initial and boundary conditions are:


$$\begin{array}{ccc} \begin{array}{ccc} & u(y,z,0)=0,\;T(y,z,0)=0,\;(y,z)\in [0,\infty )\times [0,1], & \\ & u(0,z,t)=0,\;\frac{\partial T(0,z,t)}{\partial y}=-\gamma (1+T(0)),\;t>0,\;z\in [0,1],\\ & u(y,0,t)=0,\;T(y,0,t)=0,\;t>0,\;y\in [0,\infty ),\\ & u(y,1,t)=0,\;T(y,1,t)=0,\;t>0,\;y\in [0,\infty ),\\ & u(y,z,t)\to 0,\;T(y,z,t)\to 0,\;t>0,\;y\to \infty ,\;z\in [0,1]. \end{array} & & \end{array}$$ (22)

Note: The mathematical expressions of nanofluid properties are presented in Table 1 and the thermo–physical properties of nanoparticles and base fluid are presented in Table 2.

**Table 1:** Mathematical expression of nanofluid properties [5].

Properties	Mathematical expressions
Viscosity	$\mu_{nf}=\mu_{f}{\left(1-\varphi \right)}^{-2.5}$
Density	$\rho_{nf}=(1-\varphi )\rho_{f}+\varphi \rho_{p}$
Heat capacitance	${\left(\rho C_{p}\right)}_{nf}=(1-\varphi ){\left(\rho C_{p}\right)}_{f}+\varphi {\left(\rho C_{p}\right)}_{p}$
Thermal conductivity	$\frac{k_{nf}}{k_{f}}=\frac{\left(k_{p}+2k_{f})+2\varphi (k_{p}-k_{f}\right)}{\left(k_{p}+2k_{f})-\varphi (k_{p}-k_{f}\right)}$

**Table 2:** Thermo–physical properties of mineral oil and nanoparticles [5].

Materials	Mineral oil	Al_2_O_3_
*ρ*(kg/m^3^)	861	3970
*k*(W/mK)	0.157	40
*C_p_*(J/kgK)	1860	765
*μ* (Pa.s)	0.01335	–

## Numerical analysis

Let $\varrho_{i,j}^{k+1}$ and $\vartheta_{i,j}^{k+1}$ be the numerical solutions at $\left(y_{i},z_{j},t_{k}\right)$. Define $y_{i}=i\Delta y,\;i=1,2,\cdots \;,p,z_{j}=j\Delta z,\;j=1,2,\cdots \;,q,\;t_{k}=k\Delta t,\;k=0,1,\cdots \;,n$, where $\Delta y=y_{max}∕p$, *Δz* = 1 ∕ *q* are the mesh size and $\Delta t=t_{f}∕n$, is the time step. Then we can define the following approximations

(i) for $0<k<n,\;t_{k}\leq s<t_{k+1}$:


$$\begin{array}{ccc} \frac{\partial \varrho }{\partial t}(y,z,s)≃\frac{\partial \varrho }{\partial t}(y_{i},z_{j},t_{k+1})≃\frac{\varrho_{i,j}^{k+1}-\varrho_{i,j}^{k}}{\Delta t}. & & \end{array}$$ (23)

$$\begin{array}{ccc} \frac{\partial^{2}\varrho }{\partial y^{2}}(y,z,s)≃\frac{\partial^{2}\varrho }{\partial y^{2}}(y_{i},z_{j},t_{k+1})≃\frac{\varrho_{i+1,j}^{k+1}-2\varrho_{i,j}^{k+1}+\varrho_{i-1,j}^{k+1}+\varrho_{i+1,j}^{k}-2\varrho_{i,j}^{k}+\varrho_{i-1,j}^{k}}{2\Delta y^{2}}. & & \end{array}$$ (24)

$$\begin{array}{ccc} \frac{\partial^{2}\varrho }{\partial z^{2}}(y,z,s)≃\frac{\partial^{2}\varrho }{\partial y^{2}}(y_{i},z_{j},t_{k+1})≃\frac{\varrho_{i,j+1}^{k+1}-2\varrho_{i,j}^{k+1}+\varrho_{i,j-1}^{k+1}+\varrho_{i,j+1}^{k}-2\varrho_{i,j}^{k}+\varrho_{i,j-1}^{k}}{2\Delta z^{2}}. & & \end{array}$$ (25)

(ii) fractional order derivative $\partial^{\alpha }∕\partial t^{\alpha }$ for 0<*α*<1 using *L*_1_ algorithm:


$$\begin{array}{ccc} \frac{\partial^{\alpha }\varrho }{\partial t^{\alpha }}≃\frac{1}{\Gamma (1-\alpha )}\overset{k}{\underset{s=0}{\sum }}\frac{\varrho_{i,j}^{s+1}-\varrho_{i,j}^{s}}{\Delta t}\int_{s\Delta t}^{(s+1)\Delta t}{\left(t_{k+1}-\varepsilon \right)}^{-\alpha }d\varepsilon ,\;\text{for all}\;0\leq k<n. & & \end{array}$$ (26)

Substituting $t_{k+1}-\varepsilon =\varsigma$ and changing the summation index *s* = *k* − *m* yields:


$$\begin{array}{ccc} \frac{\partial^{\alpha }\varrho }{\partial t^{\alpha }}≃\frac{1}{\Gamma (1-\alpha )}\overset{k}{\underset{m=0}{\sum }}\frac{\varrho_{i,j}^{k+1-m}-\varrho_{i,j}^{k-m}}{\Delta t}\int_{(k-m)\Delta t}^{(k+1-m)\Delta t}\varsigma^{-\alpha }d\varsigma . & & \end{array}$$ (27)

Evaluating the integral results in:


$$\begin{array}{ccc} \begin{array}{c} \frac{\partial^{\alpha }\varrho }{\partial t^{\alpha }}≃\frac{\Delta t^{-\alpha }}{\Gamma (2-\alpha )}[\varrho_{i,j}^{k+1}-\overset{k}{\underset{m=1}{\sum }}(a_{m-1}-a_{m})\varrho_{i,j}^{k-m}], \end{array} & & \end{array}$$ (28)

where $a_{m}={\left(m+1\right)}^{1-\alpha }-{\left(m\right)}^{1-\alpha },\;0\leq m\leq n$.

Exploiting aforementioned approximations in Eqs (19) and (20), we arrived


$$\begin{array}{ccc} \begin{array}{ccc} & \chi_{1}(1+\lambda_{1}^{\alpha }\frac{\Delta t^{-\alpha }}{\Gamma (2-\alpha )})[\frac{u_{i,j}^{k+1}-u_{i,j}^{k}}{\Delta t}]=\frac{p_{0}}{2}[H(t_{k})+H(t_{k+1})+\lambda_{1}^{\alpha }\frac{t_{k}^{-\alpha }+t_{k+1}^{-\alpha }}{\Gamma (1-\alpha )}] & \\ & +\chi_{2}(1+\lambda_{2}^{\beta }\frac{\Delta t^{-\beta }}{\Gamma (2-\beta )})[\frac{u_{i+1,j}^{k+1}-2u_{i,j}^{k+1}+u_{i-1,j}^{k+1}+u_{i+1,j}^{k}-2u_{i,j}^{k}+u_{i-1,j}^{k}}{2\Delta y^{2}}\\ & +\frac{u_{i,j+1}^{k+1}-2u_{i,j}^{k+1}+u_{i,j-1}^{k+1}+u_{i,j+1}^{k}-2u_{i,j}^{k}+u_{i,j-1}^{k}}{2\Delta z^{2}}]-\frac{\chi_{2}\lambda_{2}^{\beta }\Delta t^{-\beta }}{\Gamma (2-\beta )}\overset{k}{\underset{m=1}{\sum }}(d_{m-1}\\ & -d_{m})[\frac{u_{i+1,j}^{k+1-m}-2u_{i,j}^{k+1-m}+u_{i-1,j}^{k+1-m}}{2\Delta y^{2}}+\frac{u_{i,j+1}^{k+1-m}-2u_{i,j}^{k+1-m}+u_{i,j-1}^{k+1-m}}{2\Delta z^{2}}]\\ & -\frac{\chi_{2}\lambda_{2}^{\beta }\Delta t^{-\beta }}{\Gamma (2-\beta )}\overset{k-1}{\underset{m=1}{\sum }}(d_{m-1}-d_{m})[\frac{u_{i+1,j}^{k-m}-2u_{i,j}^{k-m}+u_{i-1,j}^{k-m}}{2\Delta y^{2}}+\frac{u_{i,j+1}^{k-m}-2u_{i,j}^{k-m}+u_{i,j-1}^{k-m}}{2\Delta z^{2}}]\\ & +\frac{\chi_{1}\lambda_{1}^{\alpha }\Delta t^{-(\alpha +1)}}{\Gamma (2-\alpha )}\overset{k}{\underset{m=1}{\sum }}(b_{m-1}-b_{m})[u_{i,j}^{k+1-m}-u_{i,j}^{k-m}]. \end{array} & & \end{array}$$ (29)

$$\begin{array}{ccc} \begin{array}{ccc} \chi_{3}Pr\frac{T_{i,j}^{k+1}-T_{i,j}^{k}}{\Delta t} & =(\chi_{4}+Rd)\frac{T_{i+1,j}^{k+1}-2T_{i,j}^{k+1}+T_{i-1,j}^{k+1}+T_{i+1,j}^{k}-2T_{i,j}^{k}+T_{i-1,j}^{k}}{2\Delta y^{2}} & \\ & +\frac{T_{i,j+1}^{k+1}-2T_{i,j}^{k+1}+T_{i,j-1}^{k+1}+T_{i,j+1}^{k}-2T_{i,j}^{k}+T_{i,j-1}^{k}}{2\Delta z^{2}}\\ & +\frac{E}{2\Delta y}[\tau_{xy}(k+1)(u_{i+1,j}^{k+1}-u_{i,j}^{k+1})+\tau_{xy}(k)(u_{i+1,j}^{k}-u_{i,j}^{k})]\\ & +\frac{E}{4\Delta z}[\tau_{xz}(k+1)(u_{i,j+1}^{k+1}-u_{i,j-1}^{k+1})+\tau_{xz}(k)(u_{i,j+1}^{k}-u_{i,j-1}^{k})]. \end{array} & & \end{array}$$ (30)

**Fig 2:**
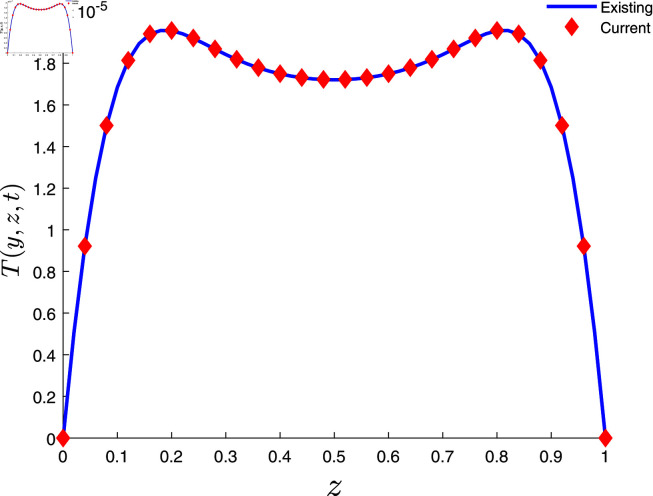
Comparison of current results with existing literature.

The discrete equations Eqs (29) and (30) are further solved using MATLAB software. The numerical method is validated by comparing the current results with previously published work [27] for limited case, see Fig 2.

## Results and discussion

This part is dedicated to investigating the effects of several physical factors on the Odroyd–B fluid occupying space over a horizontal plate. The physical factors considered in this research include fractional exponents *α*, *β*, relaxation time *λ*_1_, retardation time *λ*_2_, nanoparticle volume fraction *φ*, thermal radiation *Rd*, dissipation parameter *E*, and conjugate heat parameter *γ*. Furthermore, the obtained results are explained in depth for the parameter ranges: 0 . 1 ≤ *α* ≤ *β* ≤ 0 . 8, $0.1\leq \lambda_{2}\leq \lambda_{1}\leq 0.7$, 0 ≤ *φ* ≤ 0 . 04, 0 ≤ *Rd* ≤ 0 . 8, 0 . 1 ≤ *E* ≤ 0 . 8, and 0 . 1 ≤ *γ* ≤ 0 . 6.

**Fig 3:**
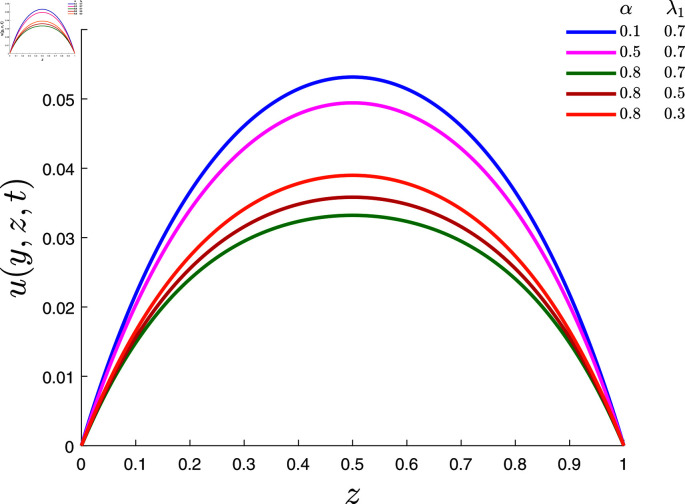
Velocity field for several values of *α* and $\lambda_{1}$.

Begin with the Fig 3 which explains the impact of fractional exponents *α* and the relaxation time $\lambda_{1}$ on the velocity field *u* when *β* = 0 . 8, $\lambda_{2}=0.1$, *φ* = 0 . 02, *Rd* = 0 . 2, *E* = 0 . 1 and *γ* = 0 . 2. It has been established that fractional exponents have a significant influence on the velocity field. As the value of *α* rises, the magnitude of the velocity diminishes as seen in Fig 3. However, a rising velocity behavior is evident with increasing values of relaxation time $\lambda_{1}$.

**Fig 4:**
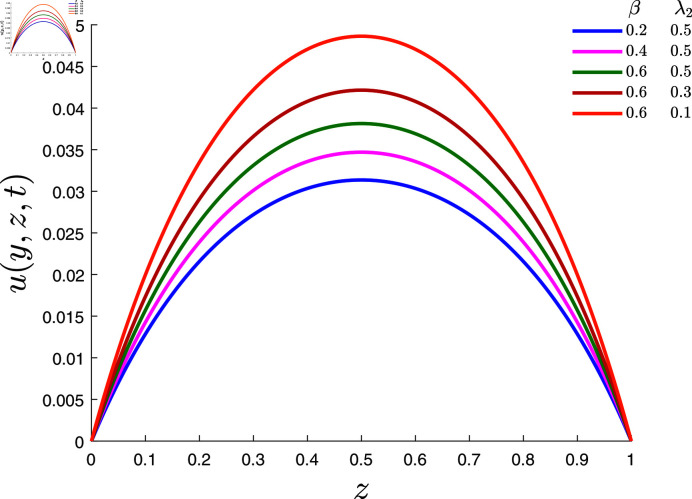
Velocity field for several values of *β* and $\lambda_{2}$.

Fig 4 shows how the velocity is affected by the fractional exponent *β* and the retardation time $\lambda_{2}$ parameters when *α* = 0 . 1, $\lambda_{1}=0.7$, *φ* = 0 . 02 , *Rd* = 0 . 2, *E* = 0 . 1 and *γ* = 0 . 2. It is obvious that raising the power index *β* increases the velocity, whereas growing the values of the time retardation parameter $\lambda_{2}$ decreases the velocity.

**Fig 5:**
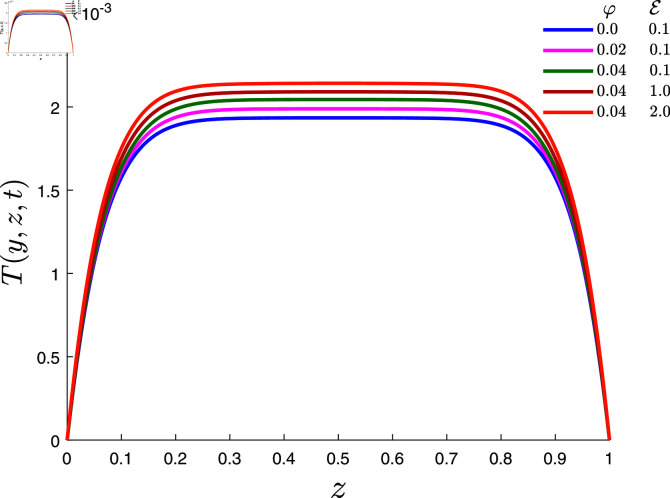
Temperature profile for several values of *φ* and *E.*

The temperature profile for various values of dissipation parameter *E* and nanoparticle volume fraction *φ* is plotted in Fig 5 when *α* = 0 . 1, *β* = 0 . 8, $\lambda_{1}=0.7$, $\lambda_{2}=0.1$, *Rd* = 0 . 2 and *γ* = 0 . 2. This figure shows that the temperature of the fluid increases as *E* increases. The fundamental reason for this is that when *E* rises, higher viscous dissipation occurs, in which mechanical energy is transformed into internal energy, causing the fluid temperature to increase. Moreover, this also figure shows an increasing trend in temperature for the accumulated values of *φ*. This behavior was due to the high thermal conductivity of the nanoparticles.

**Fig 6:**
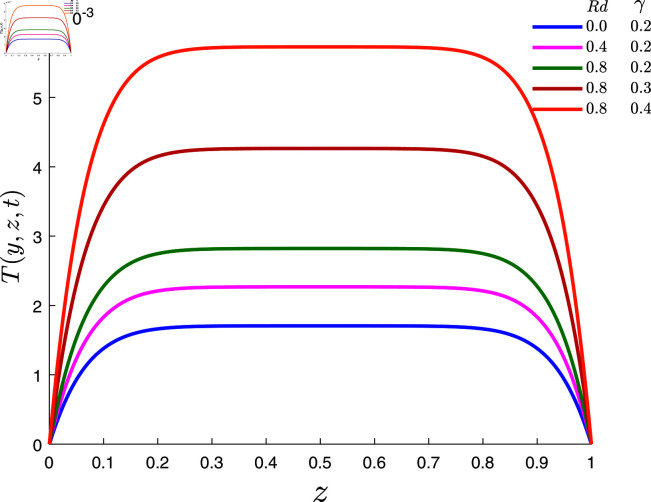
Temperature profile for several values of *Rd* and *γ.*

Fig 6 represents effects of thermal radiation *Rd* and thermal slip parameter *γ* on temperature when *α* = 0 . 1, *β* = 0 . 8, $\lambda_{1}=0.7$, $\lambda_{2}=0.1$, *φ* = 0 . 02 and *E* = 0 . 1. The results revealed that the temperature of the fluid increases for higher values of *Rd* and *γ*.

**Fig 7:**
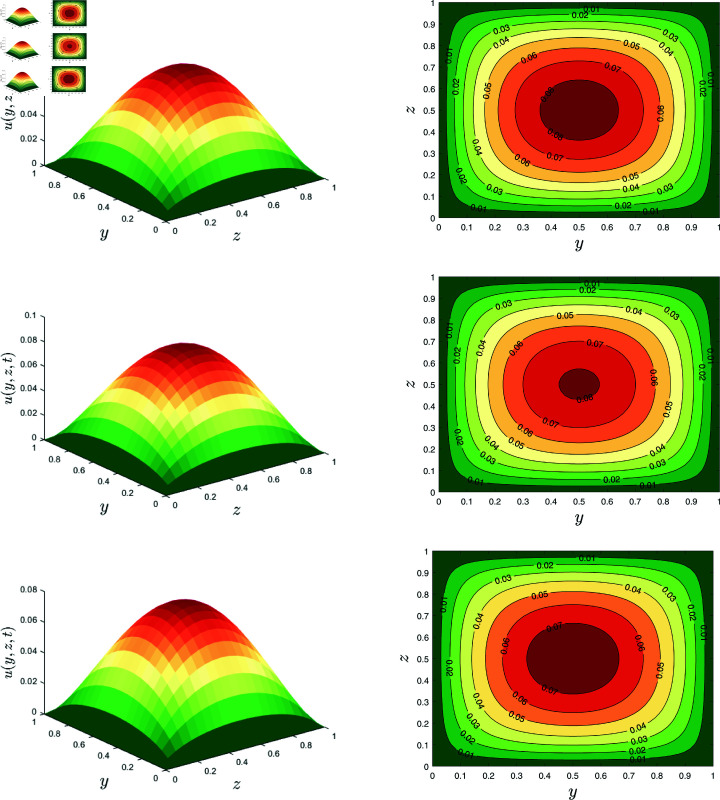
Velocity field for *φ* = 0 , 0 . 02 , 0 . 04.

In Fig 7, the surface plots(left) and contour lines (right) for the velocity are depicted. This figure shows a relationship between velocity *u* and the nanoparticle volume fraction *φ* when *α* = 0 . 1, *β* = 0 . 8, $\lambda_{1}=0.7$, $\lambda_{2}=0.1$, *Rd* = 0 . 2, *E* = 0 . 1 and *γ* = 0 . 2. A monotonic decrease in velocity with the enhancement of nanoparticle volume fraction *φ* is attributed. As the nanoparticles’ volume percentage grows, the particles’ interactions become more significant. These interactions cause drag forces inside the fluid and slow down the fluid.

**Fig 8:**
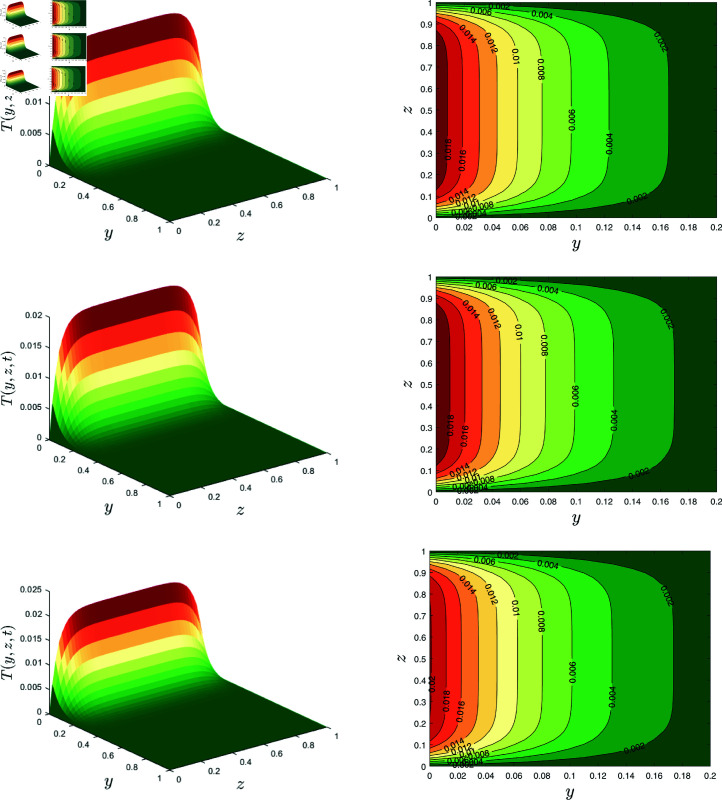
Temperature profile for *φ* = 0 , 0 . 02 , 0 . 04.

The surface and contour plots in Fig 8 present the temperature variations for different nanoparticle volume fraction values *φ* when *α* = 0 . 1, *β* = 0 . 7, $\lambda_{1}=0.7$, $\lambda_{2}=0.1,\;Rd=0.2$, *E* = 0 . 1 and *γ* = 0 . 2. These graphs demonstrate that the temperature of the fluid increased when the nanoparticle volume fraction increased.

**Fig 9:**
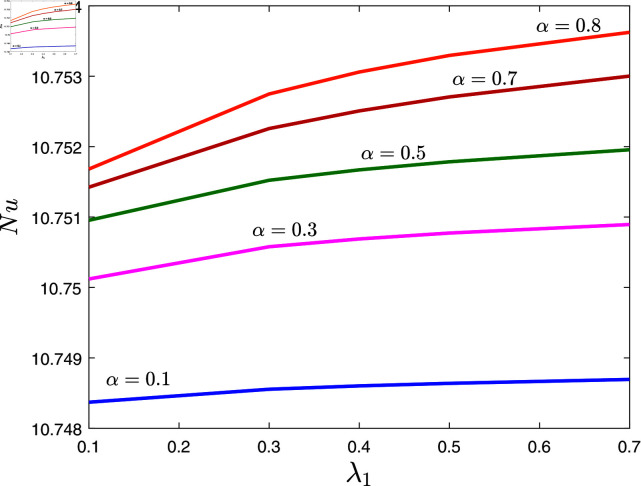
Nusselt number against $\lambda_{1}$ for several values of *α.*

The variations in the Nusselt number due to *α* and $\lambda_{1}$ are depicted in Fig 9 when $\beta =0.8,\;\lambda_{2}=0.1$, *φ* = 0 . 02 , *Rd* = 0 . 2, *E* = 0 . 1 and *γ* = 0 . 2. This figure reveals that the heat transfer rates are higher for maximum values of the fractional exponent and the relaxation time parameter.

**Fig 10:**
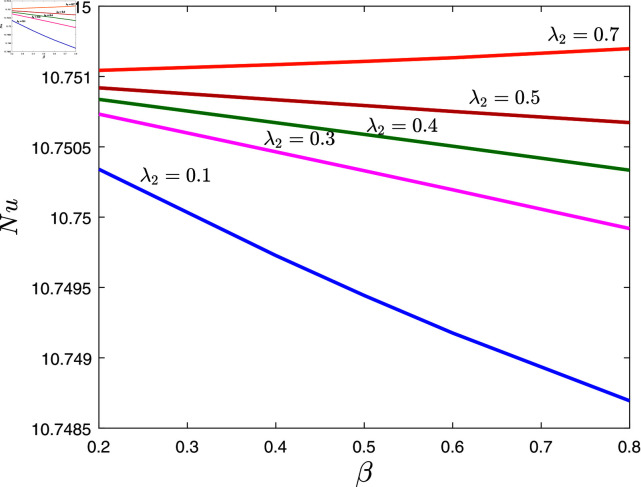
Nussetl number against *β* for several values of $\lambda_{2}$.

Fig 10 is drawn to show the effects of fractional exponent *β* and the retardation time $\lambda_{2}$ on Nusselt number when $\alpha =0.1,\;\lambda_{1}=0.7,\;\varphi =0.02,\;Rd=0.2$, *E* = 0 . 1 and *γ* = 0 . 2. In Fig 10, it is evident that when the value of *α* increases, the magnitude of the Nusselt number decreases. On the other hand, in contrast to the situation with *β*, a rising behavior of the Nusselt number is shown with increasing values of $\lambda_{2}$ in Fig 10.

**Fig 11:**
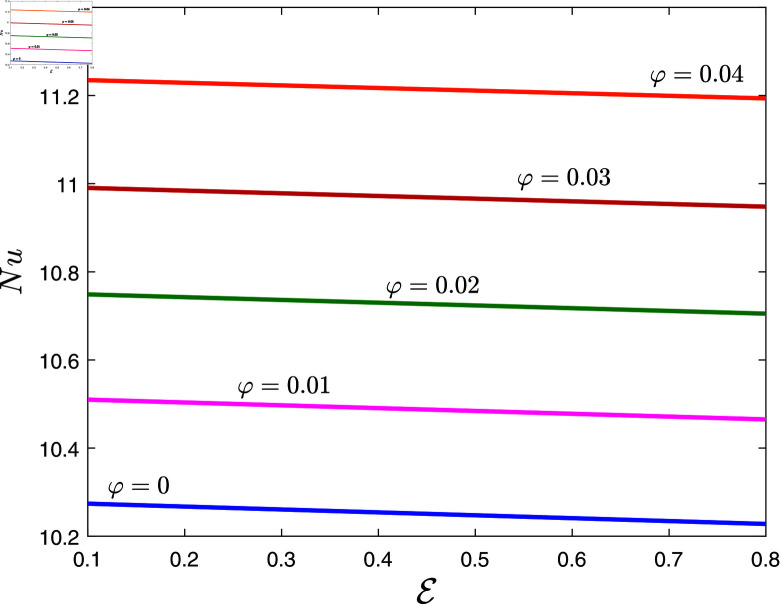
Nussetl number against *E* for several values of *φ.*

The Nusselt number *Nu* as a function of dissipation parameter *E* and nanoparticle volume fraction *φ* is illustrated in Fig 11 when *α* = 0 . 1, *β* = 0 . 8, $\lambda_{1}=0.7$, $\lambda_{2}=0.1$, *Rd* = 0 . 2 ,  and *γ* = 0 . 2. This graph shows that the heat transfer rates are significantly increased with the increase in the volume fraction of nanoparticles. It is worth mentioning that the thermal conductivity of nanoparticles is greater than that of the base fluid. Therefore, the thermal conductivity of the fluid is increased when nanoparticles are introduced to it. As a result, the nanofluid transfers heat more efficiently. Moreover, the heat transfer rate of mineral oil increased by 9.4% on adding a 4% volume fraction of nanoparticles. Note that *φ* = 0 represents mineral oil base fluid. However, the Nusselt number decreases for increasing values of *E*.

**Fig 12:**
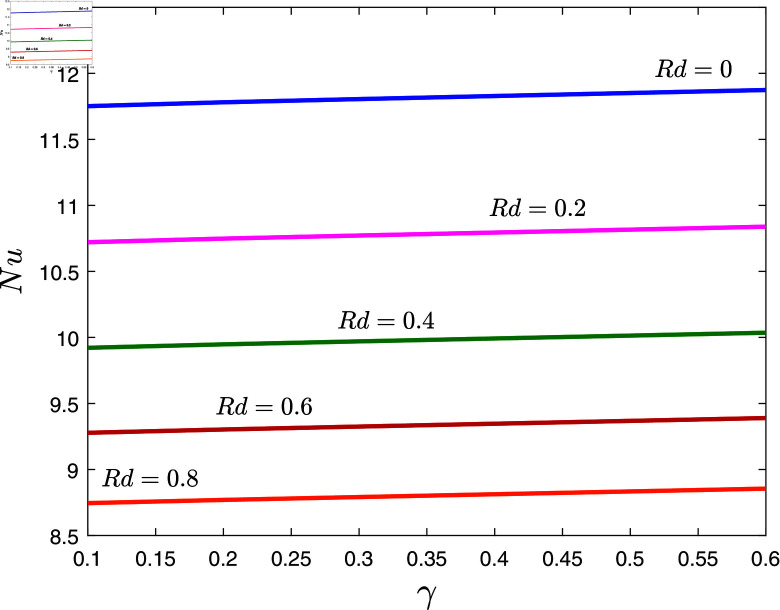
Nussetl number against *γ* for several values of *Rd.*

In Fig 12, then effects of thermal slip parameter *γ* and thermal radiation *Rd* on Nusselt number are depicted when *α* = 0 . 1, *β* = 0 . 8, $\lambda_{1}=0.7$, $\lambda_{2}=0.1$, *φ* = 0 . 02 ,  and *E* = 0 . 1. The obtained results showed that the Nusselt number decreases for increasing values of *Rd* whereas increases for increasing values of *γ*.

## Conclusions

This research analyzed heat transfer and flow of Oldroyd–B nanofluid over a horizontal plate. The non–linear fractional model is solved using the finite difference method. The integer order derivatives are integrated using the Crank–Nicolson method whereas the time fractional derivatives are evaluated using the Caputo derivative. The situations are carried out using MATLAB software. The results revealed that the relaxation time and retardation time parameters slow downs the fluid. The heat transfer rates increased with increasing values of nanoparticle volume fraction. Some major findings of the study are

- The velocity of the fluid increased and decreased against the retardation time and nanoparticle volume fraction.
- The temperature of the fluid increased due to increasing values of nanoparticle volume fraction and dissipation parameter.
- The heat transfer rates of regular fluid increased by 9.4% on adding nanoparticles to it
- The heat transfer rates increased due to relaxation and retardation time parameters.

Non–Newtonian nanofluids are used in enhanced oil recovery and drilling fluids to improve the efficiency of extraction processes. There are a few aspects of the present analysis. Although the analysis was conducted for fluid flow on a horizontal surface, it may be applied to various heat exchanger structures. The research has not considered hybrid nanofluid, which could be more beneficial in terms of applications. Therefore, heat transfer performance in a non–Newtonian fluid using hybrid nanoparticles under physical factors like magnetic field and thermal radiation, etc., is recommended.
